# Does Stimulation of Plantar Mechanoreceptors Alter Visual Spatial Localization?

**DOI:** 10.3390/jfmk11010074

**Published:** 2026-02-12

**Authors:** Philippe Villeneuve, Frédéric Viseux, Rodolfo Parreira, Maria Pia Bucci

**Affiliations:** 1 ICAR UMR 5191 CNRS, ENS de Lyon, Université Lyon 215, Parvis René Descartes PB 7000, CEDEX 07, 69342 Lyon, France; villeneuve-philippe@orange.fr; 2 Centre Hospitalier d’Hazebrouck, Service de Médecine Polyvalente, 59190 Hazebrouck, France; frederic.viseux@uphf.fr; 3 Centre Hospitalier de Saint-Amand-les-Eaux, Service de Médecine Physique et de Réadaptation Neurologique, 59230 Saint-Amand-les-Eaux, France; 4 LAMIH, CNRS, UMR 8201, Université Polytechnique Hauts-de-France, 59300 Valenciennes, France; 5 Human Movement and Reabilitation Laboratory, Universidade Evangelica de Goias, Av. Universitária, s/n-Cidade Universitária, Anápolis 75083-515, GO, Brazil; rodolfo.fcmscsp@gmail.com; 6 Neurodiderot, UMR 1141 Université Paris Cité, Hôpital Robert Debré AP-HP, 48 Bl Sérurier, 75019 Paris, France

**Keywords:** foot, oculomotor muscles, vertical heterophoria, postural control

## Abstract

**Objectives:** Few studies have previously shown an interaction between feet and gaze in postural control. The aim of the current study is to more specifically examine how vertical heterophoria (VH) varies with stimulation of the mechanoreceptors in the foot sole. A new method is proposed for fast assessment of minimum VH, especially when values are less than 1/2 diopter (pD). **Methods:** The Maddox rod test was used to assess VH with a new device made of a small point source of light located in the center of a circular box that enables the measurement of minimum VH. The VH assessment was based on a group of 95 adults in upright posture on different subtle plantar stimulations (with and without pins and on foam). **Results:** Almost half of the subjects exhibited minimum heterophoria with vertical deviations less than 1 pD, most of them (96%) with a VH ≤ 1/2 pD (small heterophoria). For the latter, a subtle plantar foot change induced by pins (1 mm high) or foam (4 mm high) can have different effects on ortho- and heterophoria. Orthophoric subjects became heterophoric with foam and pins, while heterophoric subjects became orthophoric with foam and pins, which can increase or decrease their phorias depending on the foam or pins and the type of HV. Using the new circular box makes it fast and easy to highlight even the smallest of heterophorias. We suggest that clinicians use this device to assess the effects of postural interventions, even though it has not yet proven its validity. The findings of the current study highlight the interrelation between gaze and foot systems, while the importance of the plantar mechanoreceptors is demonstrated.

## 1. Introduction

Since Magnus [1] labyrinthine, proprioceptive, visual, and plantar tactile receptors have been considered as essential factors in controlling bodily balance. Roll et al. [2] demonstrated that a stimulation of the foot muscles generates visual illusions of movement. Conversely, a stimulation of the oculomotor muscles alters the position of the center of pressure of the feet and, consequently, the plantar supports [3]. Then, Hollands et al. [4] were the first to focus on the movements of the whole body related to gaze orientation in motion. They studied the coordination of eye, head, trunk, and foot movements in a rotation task in the horizontal plane, of up to 135°. They objectified that the most important correlation was established between gaze and feet, with coordination being more significant during small rotations. The authors suggest that the superior colliculus (SC) would be responsible for whole-body shifts that relay information to the cerebellum. In a similar study using rotation up to 180°, Anastasopoulos et al. [5] showed that changes in gaze movement are influenced by the whole body, enabling it to rotate around the feet, providing a temporary gain and advantage to gain efficiency compared to segmental movements (eye/head/trunk/feet). A synchronous EMG activity between the ocular muscles and the extrinsic muscles of the feet (anterior tibialis and gastrocnemius) has also been registered. This activation of the extrinsic foot muscles precedes eye movements [6]. Based on these findings, the authors suggested that a central pattern generator located in the spinal cord synchronized rhythmic motor outputs.

Clinicians working on postural activity using the Maddox rod test (MT) [7] have reported interactions between the foot system and the visual system.

In 1890, Maddox [7], a surgeon specializing in strabismus [8], developed a test that has since become a benchmark for assessing oculomotor imbalances. These imbalances are associated with a multitude of symptoms, including blurred and double vision, eye strain, eye pain, retro-ocular pain, photophobia, headaches, motion sickness, and postural instability [9,10].

This test dissociates the vision of both eyes and makes it possible to objectify ocular deviations. To perform the MT, you need a Maddox rod and a point source of light. This test is a reference in ophthalmology because of its great simplicity [11], rapidity, and reproducibility [12,13,14].

It is worth noting that MT has also been proven helpful in posturology, for detecting minimum heterophoria (<1 pD), when standing on a foam mat, mainly vertical heterophoria (VH) in non-specific chronic pains [15,16,17] and developmental dyslexia [18,19,20]. However, the ophthalmological literature considers VH less than 0.5 diopters to be physiological [21].

The evaluation of heterophoria generally entails the utilization of a Berens prism bar, a device that facilitates the quantification of the deviation angle between the two eyes. The structure of the bar generally consisted of approximately 15 prisms of varying powers. The smaller prism (1 pD) does not permit precise evaluation of these small VH (<1 pD). A novel device was developed: a circular box that can be used to assess the smallest of heterophorias with minimal time and effort.

Since the end of the 20th century, clinical research has used the MT in an upright posture to measure horizontal [22,23] and vertical heterophorias [18,24,25,26] based on modifications related to the plantar interface.

VH represents a small vertical misalignment between the eyes, typically less than 2 prism diopters, that can lead to visual discomfort, postural imbalance, and spatial disorientation [25,26].

In contrast, horizontal phorias are more common and usually better tolerated due to more efficient horizontal fusional mechanisms [27,28]. Horizontal and vertical fusion systems can be considered as discrete entities [29].

The prevalence of clinically significant VH in the general population remains relatively low, estimated at 10–20%, but its subtle effects on postural stability and visual fatigue are increasingly recognized [27]. Desenne and Schoenstein [23] were the first to demonstrate changes in horizontal phoria of about 1 diopter (pD) after biomechanical plantar stimulation (1 mm high). Garrigues [18] also studied VH using the MT in a group of 84 children with developmental dyslexia. He assessed the children in different postures, first seated, as usual in ophthalmologic, and showed only one child orthophoric (1%). Whereas the majority of the heterophoric children (56%) became orthophoric when standing on a foam plate put under their feet.

The author suggested that these heterophorias could be explained by an integrative dysfunction in combining the proprioceptive inputs, disrupting the central regulation of visual spatial localization. These results were confirmed by Quercia et al. [20].

Loureau et al. [24] studied subjects with vertical heterophoria and assessed the potential changes in VH after inserting compressible plates with different textures, as measured by MT. They found that 80% of the subjects reduced their VH, becoming orthophoric when they were on foam with low density, both with and without reliefs. More recently, Alessandria [22] studied the effect of a thin plantar insert on horizontal heterophoria assessed with MT. The author showed that putting a 1.5 mm plantar insert under the medial to the anterior part of the calcaneus significantly changed heterophoria to orthophoria, only in the non-dominant eye. This states that minimal biomechanical stimulation of plantar cutaneous afferents could induce functional changes in oculomotor and postural organization.

For the authors, the significant reduction in VH could be related to the way information from plantar mechanoreceptors is weighted. The use of the plates may modify multisensory integration and the motor control of the postural chain linking the eyes to the feet [2]. It is also interesting to note that the physical properties of plantar interfaces interact with VH. Despite having an equivalent thickness and hardness, the higher density of the foam did not significantly affect VH [24]. Other studies have demonstrated that foot cutaneous afferents can influence visual perception. This phenomenon has been observed through the use of eye trackers [30] and the assessment of subjective visual vertical [31,32]. These interactions between foot and gaze could be explained by the fact that both of them are connected to common multiple neurological structures. Among them, the reticular formation has been demonstrated to play a pivotal role in the maintenance of postural control [33].

Postural control and ocular alignment rely on a complex integration of somatosensory, vestibular, and visual inputs to maintain spatial orientation and gaze stability [34,35]. Among these, sensory afferents from the plantar surface provide essential feedback for calibrating balance and head–eye coordination [36,37]. Alterations in foot sole stimulation can modulate postural tone and eye alignment by influencing the interaction between visual and vestibular systems [28,38]. Recent work in optometry and rehabilitation has highlighted how targeted sensory or visual feedback interventions may enhance oculomotor control and postural stability, particularly in individuals with binocular vision disorders or vestibular dysfunction [28]. This framework supports the rationale of the present study, which investigates whether different plantar stimulation conditions can induce measurable changes in VH, providing both mechanistic and clinical insights.

The plantar surface serves as a key sensory interface, contributing to postural control and body orientation. Variations in surface texture and hardness can modulate somatosensory feedback and, consequently, influence the integration of vestibular and ocular reflexes responsible for stabilizing gaze and head position [34,35]. Establishing the basis for these findings, Okubo et al. [39] first demonstrated that a reduction in postural oscillation could be achieved using small shot-gun balls emerging from a 1 mm board across the entire plantar surface.

Previous studies have demonstrated that plantar mechanoreceptors provide afferent input critical for calibrating postural equilibrium and spatial orientation [36,37,40], which interacts with visual and vestibular information to maintain body stability and oculomotor alignment [25,27,41,42].

While the impact of placing foam [43] and fine hard reliefs [39,40] under the soles of the feet on postural control is well documented, only the impact of foam on VH has been investigated [18,20,24].

Cavanaugh et al. [44] posit that the foam has the capacity to reduce pain through the activation of a central pain-modulatory system. The phenomenon of nociception inhibition may be attributable to alterations in free nerve endings. In contrast, studies have demonstrated that the application of pressure through the use of reliefs can induce a change in posture, which is a consequence of plantar mechanoreceptor stimulation [39,40]. The hypothesis that the foam has the capacity to inhibit mechanonociceptors and the relief has the capacity to stimulate mechanoreceptors suggests the possibility of two kinds of reaction on phoria.

Based on this evidence, the present study employed three plantar stimulation conditions—rigid (baseline), textured (pins), and compliant (foam)—to differentially activate plantar cutaneous receptors. The rationale for this design was to determine whether altering the somatosensory inflow from the feet would modify the distribution of vertical heterophoria (orthophoria, hypophoria, and hyperphoria), thereby revealing the contribution of plantar afferents to postural and oculomotor regulation. This experimental framework aligns with prior research showing that changes in plantar sensory input can reorganize cortical projections and modulate spatial perception [41,45].

A substantial body of research has previously been conducted on posturological pathologies using the Maddox test. The findings of this research indicated that the VH value was less than 1 pD [15,16,17,20]. The evaluation of heterophoria generally entails the utilization of a Berens prism bar, a device that facilitates the quantification of the deviation angle between the two eyes. The structure of the bar generally consisted of approximately 15 prisms of varying powers. The smaller prism (1 pD) does not permit precise evaluation of these small VH (<1 pD). A novel device was developed: a circular box that can be used to assess the smallest of heterophorias with minimal time and effort.

The primary objective of this study was to investigate how subtle stimulation of plantar mechanoreceptors affects VH in healthy adults. To achieve this, we developed a new, fast, device that enables clinicians to detect minimal VH values (<1/2 pD).

This device was used to quantify the variations in VH under three foot sole conditions—baseline, pins, and foam—chosen to modulate cutaneous afferents differently. The pins (1 mm high, spaced 1 cm apart) were designed to selectively activate mechanoreceptors such as Merkel cells and Meissner corpuscles [40] without inducing pain or instability, while the foam (4 mm thick, low density) reduces tactile feedback. This approach allowed us to examine how changes in plantar sensory input modulate oculomotor balance. Finally, although no pathological populations were included, this study may have future applications in clinical conditions characterized by sensorimotor imbalance or postural dysfunction.

## 2. Materials and Methods

### 2.1. Subjects

Ninety-five subjects: 67 females (37.58 years ± 9.13 years; body mass: 64.97 kg ± 10.45 kg; and height 165.05 ± 6.57 cm) and 28 males (36.37 years ± 6.36 years), body mass: 81.32 kg ± 14.16 kg; and height 180.37 ± 9.01 cm) participated in the study.

Subjects with head tilt, Marcus-Gunn syndrome, strabismus, and people who had undergone a strabismus surgery were excluded (i.e., 5 subjects). The assessment of subjects took place over the course of one academic year and was conducted in vocational training facilities. All subjects assigned a numerical value of zero to their pain on both the foam and 1 mm spikes, with the exception of one subject who assigned a value of 1 on the spike.

The research protocol adhered to the principles of the Declaration of Helsinki and was approved by our institutional Human Experimentation Committee (Comité Consultatif d’Éthique Local). Following a full explanation of the experimental procedure, we obtained written informed consent from all subjects involved in the study.

### 2.2. Maddox Test (MT)

The test was used to assess vertical heterophoria under different experimental conditions caused by changes in plantar afferents. In the event that the deviation is latent, it is referred to as phoria. According to Von Noorden [36], the term “phoria” (or “heterophoria”) is defined as “if one eye is excluded from participating in vision, a relative deviation of the visual axes may appear in most subjects”.

**Figure 1:**
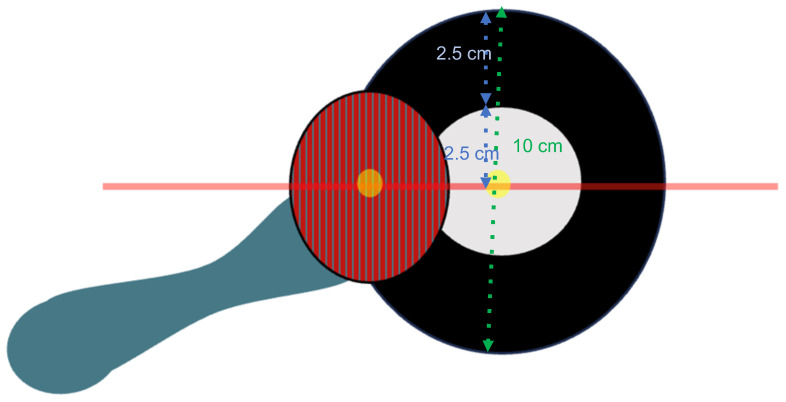
Maddox rod with red color (left-handed) positioned in front of the subjects’ left eye and the circle box. An orthophoric subject sees the horizontal line pass through the light point.

In the current study, a new device has been developed (see Figure 1) to measure vertical heterophoria (<1 pD) in a simple way to allow clinicians not trained in optics and vision to make VH evaluation easily.

The Maddox rod and a 10 cm-diameter circular box are positioned in front of the subject. The box is made of a white circle (radius = 2.5 cm) surrounded by a black ring (2.5 cm wide) with a light point (radius = 1 mm) in the center of the white circle (see Figure 1).

**Figure 2:**
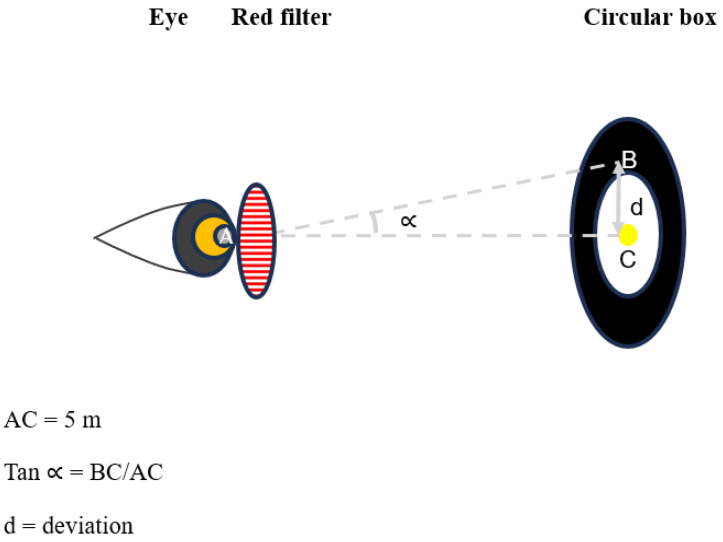
Calculation of the angle and graduation of the deviation of the visual axis measured on the circle box after the interposition of a red filter held by the examiner.

As shown in Figure 2, the deviations of the two eyes are measured by the angle ∝ that equals tan ∝ = BC/AC. The viewing distance AC is 500 cm: (1) on the yellow light: in this case, the angle ∝ is zero, (2) in the white circle: in this case, BC = 2.5 cm, (3) in the black ring: in this case, BC = 5 cm. Based on the formula from Monoyer [46], case (3), in which BC = 5 cm, angle ∝ = 0.57°, induces a deviation provided by a prism with a power of 1 pD. Based on the subject’s response, we classified phoria as orthophoria when the line is on the light point, small heterophoria < 0.5 pD when the line is in the white circle, medium heterophoria between 0.5 and 1 pD when the line is in the black ring, and large heterophoria > 1 pD when the line is outside the black ring.

### 2.3. Protocol

The subjects are in a darkened room, yet they are able to discern the circular box and the concentric circles within it. They are barefoot and focus on the light point in the center of the circular box. The subject was five meters away from the circular box, with its center, the light point, placed at the subject’s eye level. A red glass is held by the examiner in a vertical plane; otherwise, the horizontal line may appear curved. To evaluate the VH of the right eye, the experimenter stands in front of and to the right of the subject. He always begins by placing the red Maddox rod filter in front of the subject’s right eye. The process takes less than one minute. The same method is used for the left eye. Testing the right and the left eyes makes it possible to assess non-concomitant deviations [10,47].

The subject is asked to look at the light point and determine whether the red line passes through the center of the point, into the white or black circles, or outside the box.

The position of the feet is standardized. The feet rest on a 32 cm long and 26 cm wide plate, which limits variations in the feet’s spacing, rotation, or antero-posterior positioning.

### 2.4. Experimental Condition

**Figure 3:**
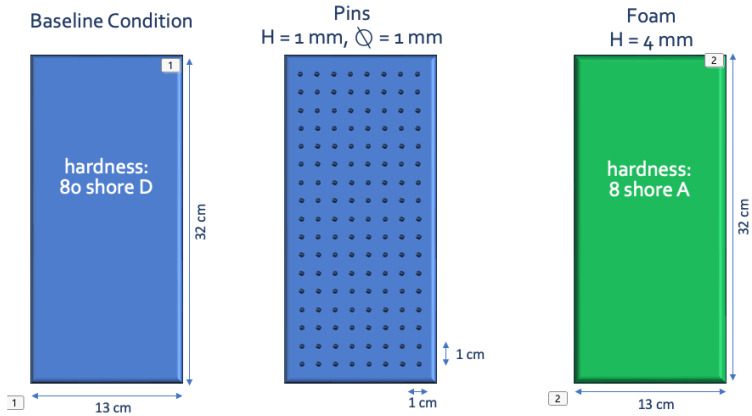
Experimental condition: Three types of plates are placed under the feet: two with non-compressible plates; one without pins (basal condition); one with pins; and one with a compressible plate (foam). Two identical plates are positioned under the feet.

To modify plantar afferents, three different conditions were conducted (see Figure 3):

(1) Okubo’s methodology (Shot-gun balls) [39] has been updated through the utilization of 3D printing technology. A non-compressible plate, made by 3D printing (Polyamide 11, density = 1.04 g/cm^3^, hardness: 80 Shore D), is put under the feet (baseline condition).
(2) The other side of the plate was covered with pins (1 mm height and diameter) spaced 1 cm apart on the entire surface of the plate.
(3) A foam under their feet. The foam (4 mm height) is made of a compressible material, Capitoner^®^ blue (Crispin Médical, La Séguinière, France), with a density of 8 Shore A, and a hardness of 220 kg/m^3^.

Prior studies on plantar mechanoreceptor stimulation had inspired the design and materials used to modify plantar afferents: additional thickness [39,40] or foam [43]. The plantar mechanoreceptor stimulation effect on balance and postural regulation was well demonstrated [34,35].

The goal was to create a controlled, reproducible method to test whether varying plantar inputs modulate vertical heterophoria.

The three experimental conditions were randomly assigned. Measurement of VH was conducted immediately after the subject stood on the plates, only once for each condition, to prevent visual adaptations [48].

### 2.5. Experimental Procedure

As stated before, VH was assessed using the Maddox test, a well-established clinical tool for detecting ocular misalignment under binocular viewing conditions. This test served as the reference standard for comparison, ensuring that the device-based measurements reflected clinically recognized phoria deviations. The MT was carried out by the same examiner during all trials following standard clinical procedures, under three plantar stimulation conditions. All three conditions were performed within a single session, with 5 min rest intervals between trials to minimize fatigue and sensory adaptation. Each test lasted about one minute.

### 2.6. Data Analysis

Depending on the results, subjects were classified as orthophoric (0), hypophoric (+), or hyperphoric (−). The measure of VH was taken in three randomized postural conditions (without stimulation, with pins, and with foam under the feet).

### 2.7. Statistical Analysis

Data normality was assessed with the Shapiro–Wilk test and found to be non-normally distributed (*p* < 0.05). Since all participants were evaluated under three related plantar stimulation conditions (baseline, pins, and foam), comparisons across conditions were performed using the Friedman test for repeated measures, followed by Wilcoxon signed-rank tests with Bonferroni correction for post hoc pairwise comparisons. For each participant, vertical heterophoria was classified into one of three categories—orthophoria, hypophoria, or hyperphoria—based on the prism deviation observed during the Maddox rod test. Here, the phoria value refers to the proportion of participants within each category under each plantar stimulation condition. It was calculated as the number of participants classified in a given VH category divided by the total number of participants (*N* = 95), expressed as a percentage. These percentage values were used as input for nonparametric statistical testing. To examine whether the distribution of vertical heterophoria classifications (orthophoria, hyperphoria, hypophoria) differed among the three conditions, we used Cochran’s Q test, as it accounts for repeated categorical data.

The significance level was set at *p* < 0.05 (two-tailed), adjusted for multiple comparisons where appropriate. All statistical analyses were processed using XLSTAT-Etudiant (2024.3.0.1423) software.

## 3. Results

**Figure 4:**
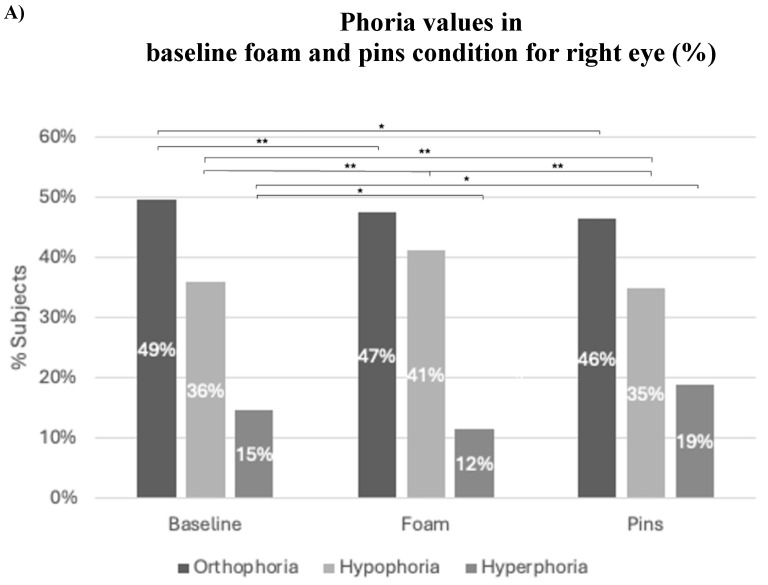

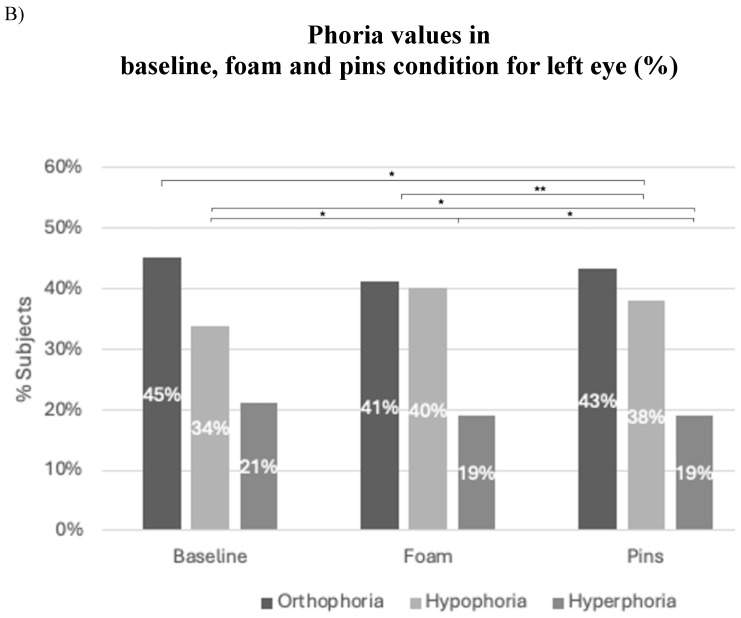
Phoria values obtained under the three conditions tested (baseline, foam, and pins) for the right eye (**A**) and the left eye (**B**), * *p* ≤ 0.05; ** *p* ≤ 0.005. Distribution of participants according to VH category: orthophoria, hypophoria, and hyperphoria under three plantar stimulation conditions: rigid, pins, and foam. The values represent the percentage of participants within each category (phoria value).

Figure 4 illustrates the distribution of participants (%) classified as orthophoric, hypophoric, or hyperphoric under plantar stimulation conditions (baseline, foam, or pins). The phoria values shown correspond to the relative percentage of subjects in each VH category.

For the right eye, among the orthophoric subjects, the percentage of orthophoria decreased significantly on foam (*p* < 0.005) and on pins (*p* < 0.05) compared to the baseline condition.

The percentage of subjects with hypophoria increased significantly (*p* ≤ 0.005) on foam, and decreased significantly (*p* < 0.05) on pins. The changes caused by foam were significantly different (*p* = 0.005) from those caused by pins.

The percentage of subjects with hyperphoria decreased significantly (*p* ≤ 0.05) on foam, and increased significantly (*p* < 0.05) on pins. However, for these subjects, the difference between foam and pins was not statistically significant.

For the left eye, among orthophoric subjects, there were no significant changes in foam or in pins compared to the baseline condition. The percentage of subjects with hypophoria on pins increased significantly (*p* ≤ 0.05). The changes induced by foam were significantly different (*p* = 0.005) from those induced by pins. The percentage of subjects with hyperphoria decreased significantly with both foam and pins (both *p* ≤ 0.05). The change induced by foam was significantly different (*p* = 0.005) from that induced by pins.

**Figure 5:**
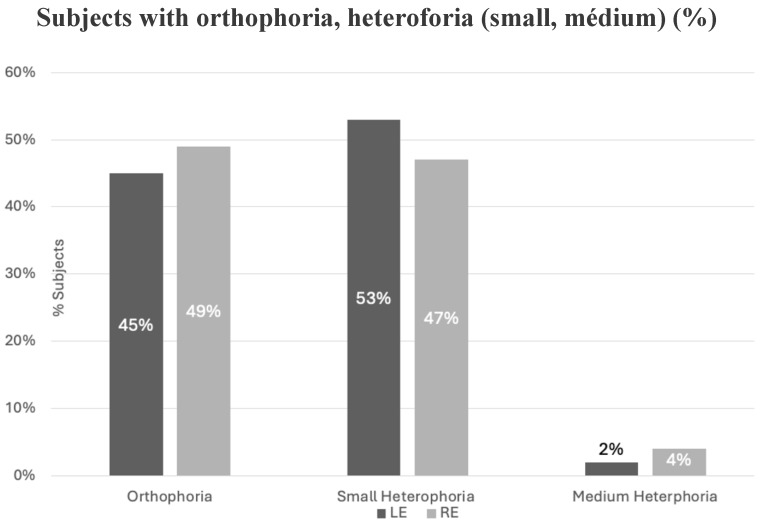
Percentage of subjects with orthophoria, small and medium heterophoria measured for each eye, respectively, during the baseline condition (LE = left eye and RE = right eye).

Figure 5 illustrates the occurrence of orthophoria as well as small and medium heterophoria measured in all subjects tested in the baseline condition for each eye. The Kruskal–Wallis test showed a significant difference in the phorias between the two eyes (*p* = 0.0001); however, comparisons between orthophoria and small and medium heterophoria, as well as between small and medium heterophoria, revealed significant differences for each eye (Dunn test, *p* < 0.0001); Bonferroni-corrected significance level: 0.0167.

## 4. Discussion

The current study aimed to explore variations in VH with the stimulation of the mechanoreceptors of the foot sole using a new device. The main findings of this study are as follows: (1) Most of the vertical heterophoric subjects (96%) show minimum heterophoria ≤ 1/2 pD (inside the white circle); (2) Vertical heterophoria changes may differ between the right and left eyes; (3) Subtle changes in plantar afferences can affect the phoria; (4) Using this new circular box makes it fast and easy to highlight even the smallest of heterophorias. These results will be discussed individually below.

(1) Most of the vertical heterophoric subjects (96%) show minimum heterophoria ≤ 1/2 pD

This study shows that nearly half of the subjects are heterophoric and have a vertical deviation of less than 1 pD (inside the box). Most of these (96%) exhibited VH ≤ 1/2 pD (inside the white circle). According to the literature on binocular vision, VH occurs in about 20% of the asymptomatic population [10], and according to van Rijn et al. [21], less than 1/2 pD indicates physiological function. This is slightly less than the eye movements when focusing on a small point (±1 pD) [49].

We can also note that approximately half of the population is orthophoric. The other half exhibits a VH deviation of less than 1 pD (within the box). No one is up to 1 pD (outside the box). The results obtained allow for the classification of the population into four distinct groups: those who see the red line in the center of the point (orthophoric), those who see either the white circle or the black circle (minimum heterophoria), and those who exhibit large heterophoria (outside the box).

(2) Vertical heterophoria changes may differ between the right and left eyes

The present study shows that vertical heterophoria changes can be very different between the right and left eyes. This change could be explained by the notion of visual dominance, which means that one eye is more used to seeing than the other. In the present study, ocular dominance was not assessed due to the absence of significant differences in ocular dominance observed in a related study on gaze alteration and foot stimulation [20].

This sighting dominance is not indicative of superior visual acuity [50]. Regarding the studies on gaze alteration and foot stimulation, Quercia et al. [20] found no significant differences between postural dysfunction and control groups with respect to ocular dominance. This study also demonstrated that tilting the head toward a shoulder resulted in a substantial change in VH for the majority of children diagnosed with developmental dyslexia. Alessandria [22] is the only one to find a dominance effect; however, he studied only subjects initially with the right dominant eye, which is different from the present study. Pepose et al. [51] demonstrated that ocular dominance will be found in 50% of subjects. Conversely, other studies conducted on emmetropic subjects have yielded no discernible ocular dominance. In the absence of pathological features, an alternating balance between both eyes is generally observed. As indicated by Mapp et al. [52] and Seijas et al. [53], this phenomenon is associated with significant variability, both between and within different types of tests.

It is important to note that the subject demonstrates significant lability in function in response to foam interposition [20,24]. This phenomenon can be explained by the concept of nociception, which was introduced by Sherrington in 1903 [54] to distinguish it from pain (algesia), since a plantar nociceptive stimulation (unconscious) has been shown to trigger withdrawal reflexes [55] and alter posture [56]. Foam interposition under the foot has been identified as a key factor in drawing attention to this phenomenon [57,58]. In the present study, only one subject reported mild pain from the pins. We can hypothesize that for some subjects, foam or pins altered the nociception.

(3) Subtle changes in the plantar afferences can alter the phoria

The current study confirms the results of previous research works on change in phoria after modifications to plantar mechanoreceptors [18,22,23,24]. It also emphasizes the importance of identifying the type of heterophoria (hypo- or hyperphoria) in each eye. The foam induces a decrease in the percentage of hyperphoric subjects in both eyes, whereas pins drive a decrease in the percentage of hypophoric subjects for the right eye and hyperphoric for the left eye.

The current findings are partially consistent with earlier observations for patients with VH [18,20,24]. The patients on foam achieved an orthophoric state. The current study for normal subjects on foam shows a decline in the percentage of hyperphoric subjects, with the majority now classified as orthophoric. However, there has been an increase in the percentage of hypophoric subjects.

However, it is important to note that in the current study, a statistically significant increase in VH was observed in the right eye of orthophoric subjects on foam (*p* < 0.005) and on pins (*p* < 0.05) compared to the baseline condition.

Although percentage changes observed (e.g., 2–3% shifts in orthophoria for the right eye) are minimal, they are clinically significant because foam or pins can induce heterophoria in a normal subject with physiologic oculomotor balance and so can be deleterious.

For the left eye, all hypophoric subjects (N = 38) increased their hypophoria on foam. Regarding the pins, there was a trend toward increased hypophoria, with the exception of two subjects who demonstrated hyperphorias. This discrepancy could explain why the percentage of subjects with pin conditions is significant, even though it is lower than for foam.

This finding is a new observation that can be explained by the fact that in an orthophoric population, the oculomotor balance is physiological; consequently, any stimulation causing a change in this balance will lead to heterophoria. According to Garrigues [18], changes in VH induced by postural stimulation may be related to the central regulation or dysregulation of visual spatial localization, which depends on the integration of postural inputs. Anastasopoulos et al. [6] showed that a proprioceptive chain involved in postural control links the eye to the foot, starting in the foot muscles. The idea that extraocular proprioception could explain the visual effects of plantar alterations is also considered [38,59,60]. In addition to visual flux cues, extraocular cues from muscular input provide information about eye movements, and as Roll et al. [3] indicated, the connection extends from oculomotor muscles to foot muscles.

The modulation of vertical heterophoria observed under different plantar stimulation conditions likely reflects the multisensory integration occurring within subcortical and cerebellar structures. The reticular formation and superior colliculi are known convergence sites for visual, vestibular, and plantar afferent inputs, contributing to gaze stabilization and head–eye coordination [33,61,62,63]. The cerebellum further refines these multisensory inputs, adjusting motor output to maintain both ocular alignment and postural balance [64,65]. Previous neurophysiological studies indicate that stimulation of plantar mechanoreceptors can influence neuronal activity in visuomotor regions such as the Edinger–Westphal nucleus and pulvinar, supporting a neuroanatomical basis for the coupling between somatosensory feedback and ocular motor control [66,67,68]. These pathways may explain the sensory-driven adjustments in phoria observed in our study.

(4) Using this new circular box makes it quick and easy to highlight even the smallest of heterophorias

Clinicians working in the postural field must be able to detect fast minimum heterophoria so that they can influence postural stability [26,69]. To diagnose heterophoria, eye specialists often use either a translucent occluder test [64] or an opaque occluder test, also known as the cover test [70,71]. However, the cover test failed to detect minimum heterophoria in normal subjects [72]. Therefore, the Maddox rod test is a more suitable option because it is simple, fast, and reproducible [11,12].

Preliminary studies have identified the minimal unphysiologic threshold measured in VH. This threshold varied from 0.3 pD with scleral coils [21], to 1 pD with MT [12], and even 2 pD [73]. Furthermore, Matheron and Kapoula [17] and Quercia et al. [20] examined patients with postural disorders who had developmental dyslexia or chronic low back, respectively, using MT. To quantify VH, they used very small prisms (0.25, 0.5, and 0.75 pD) and identified the presence of VH (<1 pD) in both populations. This value clearly separated patients from the control group. The essential feature of this VH is a liability that manifests during specific stimulation of sensory receptors involved in postural regulation [20,24]. The available evidence suggests that the small VH may have a postural origin, a hypothesis that is corroborated by the current study.

Clinical implications emphasized the value of the circular box as a practical, sensitive device to identify minimum VH, which can be associated with symptoms such as visual discomfort, headaches, balance instability, dyslexia, or chronic low back. Its ease of use supports routine screening in postural and rehabilitative contexts, potentially guiding targeted interventions that leverage plantar sensory inputs.

The observed modulation of VH under different plantar conditions may reflect the convergence of visual and somatosensory pathways within brainstem and cerebellar structures, including the superior colliculi, Edinger–Westphal nucleus, and reticular formation [33,63,66,67]. These centers are known to integrate plantar and oculomotor signals to maintain gaze stability and postural orientation. Limitations include the single measurement per condition, which may not capture intra-individual variability. Further work should explore longitudinal effects, differential responses in clinical populations, and neural pathways to correlate using neuroimaging or electrophysiological approaches.

## 5. Conclusions

The current study confirms the link between gaze and foot systems, as shown in previous research. Moreover, it demonstrates the importance of plantar mechanoreceptors. To our knowledge, this is the first study to investigate the effects of both foam and subtle pins (1 mm high) under the feet in a general population.

Furthermore, it has been demonstrated that vertical heterophoria changes may differ between the right and left eyes. A comprehensive analysis of orthophoria and the two types of heterophoria (hypo- and hyperphoria) is also provided. The results indicate that variations in phoria can vary significantly depending on the foam and pins used. In a heterophoric population, the most common outcome is a significant improvement, with heterophoric subjects becoming orthophoric when using foam and pins. Conversely, some orthophoric subjects become heterophoric when exposed to foam and pins.

Finally, the study also shows that in a general population, slightly more than half of the subjects exhibited minimal vertical heterophoria (less than 1 pD). The vast majority (96%) had small heterophoria of less than 1/2 pD.

This innovative device is particularly well-suited and useful for assessing small heterophorias, even though it has not yet proven its validity. It also makes it easier to evaluate medium and large heterophorias quickly and reproducibly. We suggest that clinicians could use this device to assess the effects of postural interventions.

## Data Availability

The datasets generated and/or analyzed during the current study are available from the author on reasonable request. The data are not publicly available due to ethical restrictions.
